# Drinking Hydrogen-Rich Water Has Additive Effects on Non-Surgical Periodontal Treatment of Improving Periodontitis: A Pilot Study

**DOI:** 10.3390/antiox4030513

**Published:** 2015-07-09

**Authors:** Tetsuji Azuma, Mayu Yamane, Daisuke Ekuni, Yuya Kawabata, Kota Kataoka, Kenta Kasuyama, Takayuki Maruyama, Takaaki Tomofuji, Manabu Morita

**Affiliations:** 1 Departments of Preventive Dentistry, Okayama University Graduate School of Medicine, Dentistry and Pharmaceutical Sciences, 2-5-1 Shikata-cho, Kita-ku, Okayama 700-8558, Japan; E-Mails: tetsuji@md.okayama-u.ac.jp (T.A.); de18053@s.okayama-u.ac.jp (M.Y.); de18019@s.okayama-u.ac.jp (Y.K.); de18017@s.okayama-u.ac.jp (K.K.); gmd421110@s.okayama-u.ac.jp (K.K.); tomofu@md.okayama-u.ac.jp (T.T.); mmorita@md.okayama-u.ac.jp (M.M.); 2 Center for Innovative Clinical Medicine, Okayama University Hospital, 2-5-1 Shikata-cho, Kita-ku, Okayama 700-8558, Japan; E-Mail: t-maru@md.okayama-u.ac.jp

**Keywords:** hydrogen, drinking water, oxidative stress, periodontitis

## Abstract

Oxidative stress is involved in the pathogenesis of periodontitis. A reduction of oxidative stress by drinking hydrogen-rich water (HW) might be beneficial to periodontal health. In this pilot study, we compared the effects of non-surgical periodontal treatment with or without drinking HW on periodontitis. Thirteen patients (3 women, 10 men) with periodontitis were divided into two groups: The control group (*n* = 6) or the HW group (*n* = 7). In the HW group, participants consumed HW 4–5 times/day for eight weeks. At two to four weeks, all participants received non-surgical periodontal treatment. Oral examinations were performed at baseline, two, four and eight weeks, and serum was obtained at these time points to evaluate oxidative stress. At baseline, there were no significant differences in periodontal status between the control and HW groups. The HW group showed greater improvements in probing pocket depth and clinical attachment level than the control group at two, four and eight weeks (*p* < 0.05). The HW group also exhibited an increased serum level of total antioxidant capacity at four weeks, compared to baseline (*p* < 0.05). Drinking HW enhanced the effects of non-surgical periodontal treatment, thus improving periodontitis.

## 1. Introduction

Periodontitis is a chronic disease of the supporting tissues of teeth, characterized by gingival bleeding, periodontal pocket formation, connective tissue destruction and alveolar bone loss [1]. Subgingival plaque biofilm is responsible for the initiation and progression of periodontitis [2,3]. Abnormal immune responses to bacterial pathogens are also accepted as the risk factor for progression of periodontitis [4].

Reactive oxygen species (ROS), including superoxide, hydrogen peroxide, and hydroxyl anions, are products of normal immune responses against bacterial pathogens. However, overproduction of ROS induces oxidative stress and such conditions damage DNA, proteins and lipids in host tissues [5,6]. There was a close relationship between oxidative stress and chronic inflammation. In dentistry, clinical studies have revealed that periodontitis is positively correlated with increased lipid, DNA, and protein oxidation in gingival crevicular fluid, saliva, or blood [7,8,9,10,11,12,13]. These indicate that oxidative stress is involved in the pathogenesis of periodontitis. Thus, a reduction of oxidative stress by consuming antioxidants would be of potential therapeutic value in the improvement of periodontitis.

Molecular hydrogen has been identified as one of the antioxidants that can reduce oxidative stress [14,15]. Drinking water containing a therapeutic dose of hydrogen (hydrogen-rich water, HW) represents an easily translatable method of delivery of molecular hydrogen. In a previous study, we demonstrated that drinking HW suppressed ligature-induced periodontitis in rats [16]. However, it is still unclear whether HW offers clinical benefits on periodontitis in humans. In the present study, we hypothesized that a systemic increase in antioxidants following drinking HW might improve the effects of periodontal treatment in humans. The purpose of this pilot study was, therefore, to compare the effects of non-surgical periodontal treatment in periodontitis patients who did and did not drink HW.

## 2. Experimental Section

### 2.1. Study Population

Thirteen volunteers (3 women, 10 men, 22–40 years old) with periodontitis were recruited from the Department of Preventive Dentistry, Okayama University Hospital between April 2013 and June 2013. All participants had bleeding on probing (BOP) ≥ 10%, one or more sites exhibiting probing pocket depth (PPD) ≥ 4 mm or clinical attachment level (CAL) ≥ 3 mm, and visible dental plaque. They were systemically healthy, and the exclusion criteria were those with <20 teeth, current smokers, pregnant women, users of antioxidant supplements, users of anti-inflammatory medicine and/or those with acute periodontitis within the previous 3 months. This study was approved by the Ethics Committee of Okayama University Graduate School of Medicine, Dentistry and Pharmaceutical Sciences (number: 1540). After obtaining informed consent, participants who fulfilled the study requirements were enrolled.

### 2.2. Study Design

At baseline, the participants were randomly assigned to the control group (*n* = 6) or the HW group (*n* = 7). The HW group drank 200–300 mL of HW every time, 4–5 times/day for a total 1000 mL from baseline to week 8. HW was made by placing a metallic magnesium stick (Doctor SUISOSUI^®^, Friendear, Tokyo, Japan) in drinking water resulting in the following chemical reaction; Mg + 2H_2_O → Mg(OH)_2_ + H_2_ [17]. All the patients received oral hygiene instruction at baseline. Both the groups received non-surgical periodontal treatment. Clinical data and serum samples were obtained at baseline, week 2 (pre-periodontal treatment), week 4 (post-periodontal treatment), and week 8 (maintenance).

### 2.3. Non-Surgical Periodontal Treatment

Non-surgical periodontal treatment included supra/sub-gingival debridement and scaling and root-planing (SRP) of all pockets (≥4 mm) with periodontal curettes (BioGent Curette, Hu-Friedy, Chicago, IL, USA) and ultrasonic scaler (Cavitron^®^, Ultrasonics, Long Island City, NY, USA) [18]. The treatment was given twice, at 2-week and 4-week stage, by an experienced dentist (Kenta Kasuyama).

### 2.4. Oral Examination

One dentist (Tetsuji Azuma) who was blinded to the treatment, performed the following oral examination. PPD and CAL were determined at six sites (mesio-buccal, mid-buccal, disto-buccal, mesio-lingual, mid-lingual and disto-lingual) on all teeth using a color-coded probe (CP-11 Color-Coded Probe, Hu-Friedy). Sites that bled upon gentle probing (25 g probing force) were recorded, and the proportion of sites with BOP and the number of BOP-positive teeth were measured in each subject. Plaque levels (PI) were measured after erythrosine staining, and were recorded with respect to their relative location to the gingival margin at four sites (mesial, distal, buccal and lingual) around each tooth [19].

### 2.5. Blood Sampling

Blood samples were obtained from the fingertip and immediately placed on ice. Following centrifugation at 3000× *g* for 5 min, the levels of reactive oxygen metabolites (ROM) and total antioxidant capacity were measured in the obtained serum samples. Measurements of ROM and total antioxidant capacity were performed immediately following blood sampling.

### 2.6. Measurements of Serum ROM and Total Antioxidant Capacity

Serum levels of ROM and total antioxidant capacity (OXY-adsorbent test; OXY) were determined using a free radical evaluator (Free Radical Elective Evaluator, Diacron International, Grosseto, Italy), in accordance with previously reported procedures [20,21]. The results of serum ROM are expressed in arbitrary units (CARR U; derived from the name of the chemist (Carratelli) who invented the test), with 1 CARR U being equivalent to 0.08 mg/dL hydrogen peroxide. Based on the manufacturer’s instructions, the following classifications were made: Normal, 250 to 300 CARR U; borderline, 301 to 320 CARR U; slight oxidative stress, 321 to 340 CARR U; oxidative stress, 341 to 400 CARR U; high oxidative stress, 401 to 500 CARR U; and very high oxidative stress, >500 CARR U. Those of total antioxidant capacity were expressed as micromoles of HClO consumed by 1 mL of sample (µmol HClO/mL). The intra-assay and interassay coefficients of variation for these assays were <5%.

### 2.7. Statistical Analysis

Fisher’s exact test and the Mann-Whitney *U* test were used to assess significant differences in clinical and biochemical parameters between the control group and the HW group. Wilcoxon signed-rank test was also used to assess significant differences in clinical and biochemical parameters between baseline and each examination. Analyses were performed using the statistical package SPSS statistics 20 (IBM Japan, Tokyo, Japan). Differences were considered significant at *p* < 0.05.

## 3. Results

At baseline, there were no significant differences in clinical and biochemical parameters except the age between the control and HW groups (Table 1). Although half of the control group consisted of females, no significant differences in any of the parameters were observed between the female and male participants.

**Table 1:** Clinical and serum parameters at baseline.

Parameters	Control Group (*n* = 6)	HW Group (*n* = 7)
Gender (male) *	3 (50.0)	7 (100.0) ^‡^
Age (years) ^†^	23 (22, 23.25)	26 (23, 30) ^§^
BMI ^†^	20.3 (19.0, 22.8)	21.7 (19.5, 24.8)
Periodontal conditions ^†^		
PPD (mm)	1.87 (1.68, 2.04)	2.20 (1.69, 2.27)
CAL (mm)	1.87 (1.68, 2.04)	2.21 (1.69, 2.27)
BOP (%)	11.1 (5.4, 30.4)	20.8 (8.3, 38.7)
PI	0.2 (0.1, 0.4)	0.2 (0.1, 1.3)
Serum oxidative stress ^†^		
ROM (CARR U)	294 (248, 371)	318 (270, 377)
OXY (µmol/mL)	398 (363, 418)	431 (379, 448)

* *n* [%]; ^†^ median [25%, 75%]; ^‡^ *p* < 0.05, compared with the control group, Fisher’s exact test. ^§^ *p* < 0.05, compared with the control group, Mann-Whitney *U* test. BMI, Body Mass Index; PPD, probing pocket depth; CAL, clinical attachment level; BOP, bleeding on probing; PI, plaque levels; ROM, reactive oxygen metabolites; OXY, OXY-adsorbent test; HW, hydrogen-rich water.

The HW group showed decreases of PPD, CAL, and BOP at week four and eight, compared to baseline (*p* < 0.05) (Table 2). The decrease of PI in the HW group was also found at week four. On the other hand, all clinical parameters in the control group did not change during the experimental period. With regard to the changes in PPD and CAL, there were significant differences between the control and HW groups at week two, four and eight (*p* < 0.05). In the HW group, PPD reduction was 1.29 mm and attachment gain was 1.30 mm, where the initial PPD was 4–6 mm. In addition, changes in CAL during the experimental period did not vary with the differences in gender and age (Table 3).

**Table 2:** Changes in clinical parameters at week 2, 4 and 8 (median (25%, 75%)).

Parameters	Control Group (*n* = 6)	HW Group (*n* = 7)
PPD (mm)		
Week 2	0.12 (0.08, 0.20) *	−0.10 (−0.31, −0.01) ^‡^
Week 4	0.12 (0.07, 0.18)	−0.28 (−0.49, −0.02) ^†,‡^
Week 8	0.01 (−0.05, 0.14)	−0.34 (−0.52, −0.11) ^†,‡^
CAL (mm)		
Week 2	0.12 (0.08, 0.20)	−0.10 (−0.31, −0.01) ^‡^
Week 4	0.12 (0.07, 0.18)	−0.28 (−0.49, −0.02) ^†,‡^
Week 8	0.01 (−0.05, 0.14)	−0.34 (−0.52, −0.11) ^†,‡^
BOP (%)		
Week 2	0.00 (−5.06, 5.08)	−2.39 (−13.01, 2.38)
Week 4	1.79 (−14.14, 3.89)	−11.90 (−24.31, 2.38) ^†^
Week 8	0.01 (−15.18, 2.67)	−5.96 (−24.41, −0.60) ^†^
PI		
Week 2	0.00 (−0.04, 0.07)	0.07 (−0.13, 0.46)
Week 4	−0.02 (−0.19, 0.25)	−0.17 (−0.65, 0.00) ^†^
Week 8	0.09 (−0.07, 0.13)	−0.03 (−0.72, 0.25)

* Differences (each time point minus baseline); ^†^ *p* < 0.05, compared with the baseline, using Wilcoxon signed-rank test. ^‡^ *p* < 0.05, compared with the control group, using Mann-Whitney *U* test.

**Table 3:** Changes in CAL in each individual (mm).

Patient	Gender	Age (Years)	Baseline	Week 2	Week 4	Week 8
Control group						
A	Male	22	1.78	1.90	1.93	1.79
B	Male	23	1.95	2.04	2.02	1.96
C	Male	24	2.09	2.15	2.18	2.04
D	Female	22	1.73	2.01	1.89	1.68
E	Female	23	1.54	1.66	1.79	1.70
F	Female	23	2.02	2.19	2.08	2.15
HW group						
G	Male	40	2.21	2.17	1.72	1.77
H	Male	30	2.27	2.17	1.99	1.93
I	Male	26	2.22	1.81	1.57	1.49
J	Male	25	1.64	1.47	1.42	1.46
K	Male	28	2.32	2.01	1.84	1.80
L	Male	23	1.79	1.78	1.77	1.68
M	Male	23	1.69	1.68	1.68	1.67

The control group showed a higher level of serum ROM at week eight, compared to baseline (*p* < 0.05) (Table 4). There was a significant difference in the serum level of ROM between the control and the HW groups at week eight. The HW group also exhibited a higher level of serum total antioxidants at week four, compared to baseline (*p* < 0.05).

**Table 4:** Changes in biochemical parameters at week 2, 4 and 8 (median (25%, 75%)).

Parameters	Control Group (*n* = 6)	HW Group (*n* = 7)
ROM (CARR U)		
Week 2	39 (5, 76) *	35 (3, 42)
Week 4	49 (29, 85)	32 (11, 43)
Week 8	28 (11, 106) ^†^	−15 (−67, 13) ^‡^
OXY (µmol/mL)		
Week 2	24 (11, 67)	−1 (−22, 66)
Week 4	49 (18, 70)	56 (−18, 86) ^†^
Week 8	12 (−46, 35)	15 (−43, 76)

* Differences (each time point minus baseline); ^†^ *p* < 0.05, compared with the baseline, using Wilcoxon signed-rank test. ^‡^ *p* < 0.05, compared with the control group, using Mann-Whitney *U* test.

## 4. Discussion

To the best of our knowledge, this is the first study to investigate the effects of drinking HW on periodontitis in humans. At baseline, the control and HW groups had similar periodontal status. On the other hand, the improvements of PPD and CAL were greater in the HW group than in the control group at week two, four and eight. The HW group, but not the control group, also showed improvements of PPD, CAL and BOP at week four and eight. These observations indicate that drinking HW improved the effects of non-surgical periodontal treatment in periodontitis patients.

In this study, participants were randomly assigned to the control group or the HW group. Therefore, all female participants were assigned to the control group by chance. Likewise, all participants ≥ 30-years-old were also assigned to the HW group. It is known that age and gender have an influence on periodontal status [22]. However, in our observations, changes in CAL did not differ with the age and gender. It is possible that differences in age and gender had little effect on the present periodontal status.

At week two, all participants had not received non-surgical periodontal treatment. At this point, a significant difference in PPD and CAL was already seen between the control and the HW groups. However, the HW group did not show improved PPD and CAL at week two compared to baseline. Although only drinking HW may be beneficial to the periodontal health, the combination of drinking HW and non-surgical periodontal treatment would be necessary for significant improvement of the periodontal status.

At week eight, the periodontal parameters in the control group appeared to return to baseline. In this study, only two times of periodontal treatment was carried out only twice, at two-week and four-week stage. Therefore, the improvement of periodontitis by our periodontal treatment might be transient. On the other hand, the improvement of periodontitis by periodontal treatment and drinking HW continued until week eight. Drinking HW may keep up the effects of periodontal treatment on periodontitis.

In a review, mean PPD reduction after non-surgical periodontal treatment was 1.29 mm and attachment gains was 0.55 mm where the initial PPD was 4–6 mm [23]. In our findings, mean PPD reduction in the HW group was 1.29 mm and attachment gains was 1.30 mm at week eight where the initial PPD was 4–6 mm. These indicate that CAL or PPD improvement in the HW group could be generally considered as clinically significant.

It is known that progression of periodontitis is induced by increased oxidative stress [7]. In our findings, the HW group showed an increased serum level of total antioxidants capacity at week four. The increased level of antioxidant capacity following drinking HW reduced periodontal oxidative stress [16], and that such conditions may improve the effects of non-surgical periodontal treatment in periodontitis. This concept is supported by previous studies, which demonstrate that the anti-oxidative effects of HW suppressed inflammation in the periodontal tissue [16] and the peritoneum tissue [24]. However, we found that the serum level of ROM decreased at week eight after drinking HW and non-surgical periodontal treatment, but no significant difference was detected. These observations are different from the previous results, showing that non-surgical periodontal treatment decreased plasma level of ROM [18]. In this study, the mean serum level of ROM at baseline was 316 CARR U in the control group and 324 CARR U in the HW group respectively, suggesting that both the groups had slight oxidative stress at baseline. On the other hand, in a previous study [18], the plasma level of ROM at baseline was 442 CARR U. The discrepancy between the present and previous studies could be due to blood ROM level at baseline.

The application of antioxidants could prove useful in the management of periodontal status in animal models. We had examined the effects of vitamin C on rat periodontitis model [25]. In that study, the results reveal that chronic vitamin C intake could accelerate improvement of gingival oxidative stress and periodontitis. In another study, consumption of a cocoa-polyphenol-enriched diet for four weeks suppressed polymorphonuclear leukocyte infiltration with a decreased oxidative stress in the periodontal lesion [26]. In addition, dietary supplementation of proanthocyanidins, a novel flavonoid extracted from grape seeds, elevated blood levels of nonenzymatic antioxidants and prevented cellular infiltration of inflammatory cells in the periodontal tissue at 30 days [27]. These findings in animal studies suggest that antioxidant intake is useful for modifying periodontal oxidative stress and has potential benefits for management of periodontitis, and this concept is consistent with the current study.

In human models, as in this study, the effects of antioxidant supplementation on periodontal status have been proved. The intake of grapefruit led to an increase in plasma vitamin C levels and reduced gingival bleeding in patients with periodontitis [28]. Supplementation with vitamin C in subjects with progressive periodontal disease but not chronic periodontal disease increased the number of collagen bundles in the periphery of fibroblasts and a daily intake between 30 to 50 mg of vitamin C daily was recommended [29]. A placebo-control study revealed that supplementation of 8 mg/day lycopene, an antioxidant found in tomatoes and other red fruits and vegetables, showed a significant reduction in gingival bleeding when compared to the control at two weeks [30]. Antioxidant supplementation of certain concentration may be clinically effective in suppressing the progression of periodontitis. However, effects of antioxidant supplementation on periodontitis are still not clear due to a limited number of randomized controlled trials. It is difficult to reach a definitive conclusion on the safety and efficacy of this treatment. The need for confirmatory evidence and evaluation of the safety issues seems to be an important requirement.

The relevant weakness of this study is that all subjects were recruited at the Okayama University Hospital. This may limit the ability to generalize our findings to the general population. In addition, all patients had mild periodontitis in this study. The effects of drinking HW on severe periodontitis patients may differ from our results. Furthermore, the sample size of 6–7/group is small. Large-scale clinical trials should be conducted to validate the results of this pilot study.

## 5. Conclusions

The improvements of PPD and CAL were greater in the HW group than in the control group at week two, four and eight. The HW group also increased serum level of total antioxidants capacity at week four. Drinking HW might be beneficial in improving periodontitis.
